# Interfacial construction of P25/Bi_2_WO_6_ composites for selective CO_2_ photoreduction to CO in gas–solid reactions†

**DOI:** 10.1039/d3ra00418j

**Published:** 2023-03-14

**Authors:** Daohan Liu, Minli Zeng, Zhen Li, Zhiqi Zhu, Yu Chen, Kunyapat Thummavichai, Oluwafunmilola Ola, Nannan Wang, Yanqiu Zhu

**Affiliations:** a State Key Laboratory of Featured Metal Materials and Life-cycle Safety for Composite Structures, School of Resources, Environment and Materials, Guangxi University Nanning 530004 China wangnannan@gxu.edu.cn; b College of Engineering, Mathematics and Physical Sciences, University of Exeter Exeter EX4 4QF UK Y.zhu@exeter.ac.uk; c Department of Mathematics, Physics and Electrical Engineering, Faculty of Engineering and Environment, Northumbria University NE1 8ST UK; d Advanced Materials Group, Faculty of Engineering, The University of Nottingham Nottingham NG7 2RD UK

## Abstract

Photocatalysis provides an attractive approach to convert CO_2_ into valuable fuels, which relies on a well-designed photocatalyst with good selectivity and high CO_2_ reduction ability. Herein, a series of P25/Bi_2_WO_6_ nanocomposites were synthesized by a simple one-step *in situ* hydrothermal method. The formation of a heterojunction between Bi_2_WO_6_, which absorbs visible light, and P25, which absorbs ultraviolet light, expands the utilization of sunlight by the catalysts, and consequently, leads to a remarkably enhanced CO_2_ selective photoreduction to CO. The maximum CO yield of the P25/Bi_2_WO_6_ heterojunction under simulated solar irradiation was 15.815 μmol g^−1^ h^−1^, which was 4.04 and 2.80 times higher than that of pure P25 and Bi_2_WO_6_, respectively. Our investigations verified a Z-scheme charge migration mechanism based on various characterization techniques between P25 and Bi_2_WO_6_. Furthermore, *in situ* DRIFTS uncovered the related reaction intermediates and CO_2_ photoreduction mechanism. Our work sheds light on investigating the efficacious construction of Bi_2_WO_6_-based hybrids for light-driven photocatalysis.

## Abstract 2

Proposed mechanism of CO_2_ photoreduction of the P25/Bi_2_WO_6_ nanocomposites.

## Graphical abstract

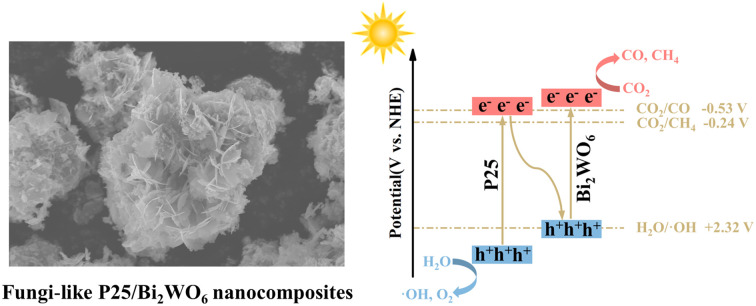

## 1 Introduction

The continuous consumption of global fossil energy and the increasing concentration of the greenhouse gas carbon dioxide (CO_2_) has led to a severe energy crisis and environmental pollution problems, which are not in line with the requirements of sustainable human development.^1–3^ Therefore, how to convert CO_2_ into chemical fuels has become a pressing problem.^4,5^ So far, CO_2_ reduction has usually been achieved by hydrogenation reactions at high temperature and pressure or in the presence of precious metal co-catalysts.^6,7^ On the other hand, inspired by plant photosynthesis, using solar energy to convert CO_2_ into useable chemicals is a low-cost and environmentally friendly strategy.^8–10^ Various photocatalysts, such as titanium dioxide (TiO_2_), cuprous oxide (Cu_2_O), carbon nitride (g-C_3_N_4_), strontium titanate (SrTiO_3_), bismuth chloride (BiOCl), and tungsten oxide (WO_3_), have been extensively studied and applied in this field of photocatalytic CO_2_ reduction.^11–16^ In general, the process of photocatalytic CO_2_ consists of the following steps. First, electron–hole pairs are excited by photons, where the photon energy is equal to or greater than the semiconductor bandgap. Second, light-induced carriers are separated and transported to the surface of the photocatalyst. Third, light-induced electrons react with CO_2_ at the surface-active center of the photocatalyst.^17^ However, the efficiency of most single-component photocatalysts for photocatalytic CO_2_ reduction is still far from practical due to the rapid compounding of electron–hole pairs and the lack of surface-active centers. In order to improve photocatalytic efficiency, one of the most effective ways is to construct heterojunction by compounding two semiconductors.^18,19^ This method can improve the separation and transfer of electron–hole pairs and provide more surface-active centers for photocatalytic reactions. Therefore, the construction of suitable heterojunction is essential to improve the CO_2_ reduction performance of photocatalysts.

TiO_2_ has the advantages of strong redox performance, non-toxic, low price, and high stability, and it has a broad development prospect in the field of photocatalytic CO_2_ reduction.^20–22^ Commercial TiO_2_ (P25) is a mixture of anatase and rutile TiO_2_ with forbidden bandwidths of 3.2 and 3.0 eV, respectively, and a wide band gap, which has solid photocatalytic properties under UV-vis light, and its particle size ranges from 20 to 40 nm, which has a large specific surface area.^23^ However, only 4% of sunlight is in the UV, and the lower light utilization and rapid compounding of electron–hole pairs limit the practical application of TiO_2_ photocatalytic CO_2_ reduction.^24,25^ Therefore, several methods have been used to improve the photocatalytic performance of TiO_2_, including doping, noble metal deposition, surface modification, and construction of heterojunction.^26–29^ As a representative method, combining UV-responsive semiconductor TiO_2_ and visible-light-responsive semiconductor to construct heterojunction facilitates carrier migration and separation. It improves the light adsorption capacity, improving its photocatalytic CO_2_ reduction performance.

Bismuth tungstate (Bi_2_WO_6_) is a visible light-responsive bismuth-based photocatalyst with a forbidden bandwidth of about 2.8 eV, one of the Aurivillius phase oxides, it is a complex, layered material consisting of alternating stacks of [Bi_2_O_2_]^2+^ layers and [WO_4_]^2−^ layers.^30^ This layered structure facilitates the separation of electron–hole pairs and confers them to a high photocatalytic activity.^31^ However, Bi_2_WO_6_ exhibited poor light absorption performance and low photoconversion efficiency at wavelengths less than 450 nm.^32^ Combining Bi_2_WO_6_ and other semiconductor materials to construct heterojunction should be an effective strategy to solve this problem. So far, heterojunction structures have attracted significant attention in photocatalysis, and many heterojunction photocatalysts have been developed, such as Bi_2_WO_6_/MoO_3_, BiSn_2_O_7_/Bi_2_WO_6_, black phosphorus/Bi_2_WO_6_, and g-C_3_N_4_/Bi_2_WO_6_, *etc.*^33–36^ Combining P25 nanoparticles with Bi_2_WO_6_ can improve the photocatalytic performance. The synergistic effect between them can improve the light adsorption ability and facilitate the separation of electron–hole pairs. More importantly, the agglomeration phenomenon of P25 nanoparticles can be effectively improved. To the best of our knowledge, the application of P25/Bi_2_WO_6_ nanocomposites in photocatalytic CO_2_ reduction has been little studied.

In this work, we have successfully prepared P25/Bi_2_WO_6_ nanocomposites using a simple one-step *in situ* hydrothermal method. It was used for the photocatalytic reduction of CO_2_ in the absence of sacrificial agents, and the physical and photochemical properties of the material were characterized by various characterization tools. The reaction process and mechanism of the photocatalytic system were further investigated by *in situ* infrared testing. Compared with pure P25 and Bi_2_WO_6_, there is a strong synergy between the nanocomposites, which not only broadens the light absorption range but also suppresses the electron–hole pair recombination and has a high selectivity for carbon monoxide (CO). This study can provide new ideas for Bi_2_WO_6_-based nanocomposites in photocatalysis.

## 2 Experimental section

### 2.1. Materials

Sodium tungstate dihydrate (Na_2_WO_4_·2H_2_O, 99.5%) and titanium dioxide (P25) were purchased from Shanghai Macklin Biochemical Co., Ltd, China. Bismuth(III) nitrate pentahydrate (Bi(NO_3_)_3_·5H_2_O, 99.0%) was purchased from Sinopharm Chemical Reagent Co., Ltd, China. Cetyltrimethylammonium bromide (CTAB, 99.0%) was purchased from Damao Chemical Reagent Factory Co., Ltd, China. Sodium hydrogen carbonate (NaHCO_3_, 99.5%) was purchased from Guangdong Guanghua Sci-Tech Co., Ltd, China. All chemicals were used as received without further purification. The water used throughout the research is deionized water.

### 2.2. Synthesis

#### 2.2.1 Preparation of the P25/Bi_2_WO_6_ photocatalyst

**Scheme 1:**
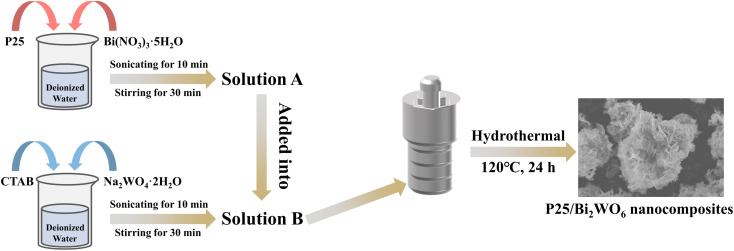
Schematic illustration of the preparation process of the P25/Bi_2_WO_6_ composite.

P25/Bi_2_WO_6_ photocatalyst was prepared according to the previous reports with some modifications.^37^ In a typical procedure (Scheme 1), 2 mmol Bi(NO_3_)_3_·5H_2_O and a known mass of P25 were mixed in 40 mL deionized water, which was labeled as Solution A. 1 mmol Na_2_WO_4_·2H_2_O and 0.05 g CTAB were added to 40 mL of deionized water with ultrasonic for 10 min and stirring for 30 min at ambient temperature, which was labeled as Solution B. Then Solution A was slowly added into Solution B and stirring for 30 min at ambient temperature. The resulting suspension was transferred into a 100 mL Teflon-lined autoclave and heated at 120 °C for 24 h. The final products were collected by centrifugation, washed several times with ethanol and deionized water, and dried at 60 °C in vacuum for 12 h. The mass ratios of P25 to Bi_2_WO_6_ were set as 10%, 20%, 30%, and 40%, and they were labeled as P25/BWO-10, P25/BWO-20, P25/BWO-30, and P25/BWO-40, respectively.

#### 2.2.2 Preparation of Bi_2_WO_6_ nanosheets

For comparison, under the same experimental procedure, the pure Bi_2_WO_6_ were synthesized without adding P25, which was labelled as BWO.

### 2.3. Characterization

Powder X-ray diffraction (XRD, Rigaku D/MAX 2500 V, Rigaku Corporation, Japan) was used to investigate the crystal phase structure. The wavelength of the copper target was *λ* = 0.15418 nm, and the working current and accelerating voltage were 150 mA and 40 kV, respectively. The appearance and micro shape of the catalysts were analyzed by scanning electron microscope (SEM, Sigma 300, Carl Zeiss, Germany) and scanning transmission electron microscope (TEM, Tecnai F20, FEI, USA), respectively. The accelerating voltages of SEM and TEM were 20 kV and 200 kV, respectively. The sample's elemental distribution was detected using energy dispersive spectroscopy (EDS) of Oxford Instrument. The chemical environments of elements on the catalyst's surface were characterized by X-ray photoelectron spectroscopy (XPS, ESCALAB 250XI, Waltham, USA). Before deconvolution, calibration was performed, where all the binding energies were charged and corrected by setting the adventitious carbon signal (C 1s) to 284.8 eV. Raman spectrometer (inVia Reflex 1500080S, Nottingham, UK) was employed to characterize the surface structure of the catalysts. Fourier Transform infrared spectroscopy (FT-IR) spectra were carried out with a Fourier transform infrared spectrometer (FT-IR, Nicolet iS50, Thermo Fisher Scientific, USA). UV-vis diffuse reflectance spectra (DRS, UV-3600Plus, SHIMADZU, Japan) were obtained to investigate the light-harvesting ability of the catalysts. Photoluminescence spectra (PL, FL3C-111 TCSPC, HORIBA, Japan) was obtained to study carriers' separation of samples.

### 2.4. Photoelectrochemical measurement

The photocatalyst samples' photoelectrochemical (PEC) properties were evaluated using an electrochemical workstation (CHI660D, Chenhua Instrument, China) with a three-electrode system. The samples coated on pure fluorine-doped tin oxide (FTO) glasses acted as working electrodes, while Pt and Ag/AgCl electrodes acted as counter and reference electrodes, respectively, with 0.5 M Na_2_SO_4_ as the electrolyte solution. A full spectrum Xe lamp (300 W, PLS-SXE300, Perfect light, China) was adopted as the light source.

### 2.5. Photocatalytic CO_2_ reduction experiments

The photocatalytic CO_2_ reduction reaction (CO_2_RR) was realized in a 100 mL confined device equipped with circulating condensate water and a 300 W Xe lamp (UV light spectrum range of 320–780 nm). In a typical process, 20 mg photocatalyst was ultrasonically dispersed into ethanol and dispensed onto a 30 mm × 30 mm flat quartz plate. After thoroughly drying the catalyst, the flat quartz plate was placed into the reactor. Then, 40 mL 0.1 M NaHCO_3_ aqueous solution was injected to react. Before irradiation, the reaction mixture was purged with bubbling CO_2_ for 40 min in the dark to ensure adsorption–desorption equilibrium was attained on the photocatalyst surface. The reactor's temperature was controlled at 25 °C by cooling water circulation. Then the reactor was continuously irradiated with UV-vis light using a 300 W Xe lamp. After an hour of reaction, the gaseous products were analyzed by a gas chromatograph (GC-2014C, SHIMADZU, Japan) equipped with a flame ionization detector (FID) and a thermal conductivity detector (TCD).

### 2.6. *In situ* diffuse reflectance infrared Fourier transform spectra (DRIFTS) measurement

*In situ* diffuse reflectance infrared Fourier transform spectra (DRIFTS) were recorded on a Fourier transform infrared spectrometer (FT-IR, Nicolet iS50, Thermo Fisher Scientific, USA). The spectra in absorbance units were acquired with a resolution of 4 cm^−1^ using 32 scans. Firstly, samples were purged with Ar for an hour, and then a mixture of CO_2_ and water vapor was taken into the chamber at 5 mL min^−1^ for 20 min. After that, samples were under illumination (300 W Xe lamp) for 40 min. The spectra were collected every 10 min.

## 3 Results and discussion

### 3.1. Structural characterization

**Fig. 1:**
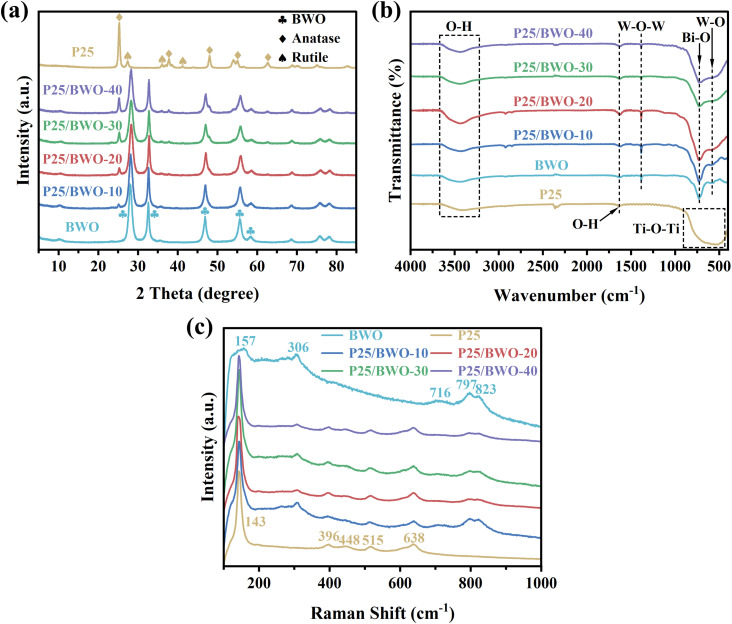
(a) XRD patterns, (b) FT-IR spectra, and (c) Raman spectra of the as-prepared samples.

The crystalline phases of P25, BWO, and P25/BWO nanocomposites with different contents were analyzed by XRD. As shown in Fig. 1a, the diffraction peaks located at 28.3°, 32.8°, 47.3°, 56.1°, and 58.4° belonged to the (131), (200), (202), (133), and (262) crystalline planes of BWO (JCPDS no. 39-0256), respectively.^38^ The diffraction peaks located at 25.3°, 37.8°, 48.0°, 55.0°, and 62.7° assigned to the (101), (004), (200), (211), and (204) crystal planes of anatase TiO_2_ (JCPDS no. 21-1272), respectively. The diffraction peaks located at 27.4°, 36.0°, and 41.2° corresponded to the (110), (101), and (111) crystal planes of rutile TiO_2_ (JCPDS no. 21-1276), respectively.^39^ The peaks of pure P25 and BWO were sharp and had high intensity, indicating that the prepared samples had good crystallinity. Only the diffraction peaks of P25 and BWO appeared in the X-ray diffractograms of all four nanocomposites. The larger the percentage of P25 content, the stronger the intensity of the characteristic peaks of P25 in the nanocomposites. The results indicated that the combination of P25 and BWO had high purity and crystallinity.

FT-IR investigated the specific functional groups of all samples. As shown in Fig. 1b, in the FT-IR spectra of BWO, the peaks at 3447 and 1636 cm^−1^ were caused by O–H vibrations. In addition, the peaks at 1389, 728, and 576 cm^−1^ corresponded to W–O–W stretching, Bi–O stretching, and W–O stretching, respectively.^40^ In the FT-IR spectra of P25, the stretching vibrational band from 850 to 433 cm^−1^ belonged to the Ti–O–Ti bond of P25.^21^ The four P25/BWO nanocomposites with different contents had both the characteristic absorption peaks of P25 and BWO. Meanwhile, their FT-IR spectra were more similar to that of BWO, which may be because BWO was the main component in the nanocomposites. The above experimental results can prove the successful synthesis of P25/BWO nanocomposites.

Raman spectra were used to investigate the structure of the samples further. Fig. 1c shows the Raman spectra of P25, BWO, and P25/BWO nanocomposites with different contents of wavenumber in the range of 100 to 1000 cm^−1^. Generally, the typical characteristic peaks of Raman spectra of BWO were located at 157, 306, 716, 797, and 823 cm^−1^. Among them, the peak at 157 cm^−1^ was attributed to the external vibration of WO_6_^6−^.^41^ The vibrational peaks in the range of 600 to 1000 cm^−1^ were attributed to the vibrational stretching mode of the W–O bond. In contrast, the vibrational peaks near 306 cm^−1^ were mainly attributed to the vibrational stretching mode between Bi^3+^ and WO_6_^6−^.^42^ The typical characteristic peaks of Raman spectra of anatase TiO_2_ were located at 143, 396, 515, and 638 cm^−1^, and rutile TiO_2_ was located at 448 cm^−1^. The O–Ti–O variable angle vibration peak with the highest intensity was 143 cm^−1^. In the Raman spectra of the four P25/BWO nanocomposites with different contents, only the typical characteristic peaks of P25 and BWO appeared. The results indicated that there was a strong interaction between P25 and BWO.

### 3.2. Morphological characterization

**Fig. 2:**
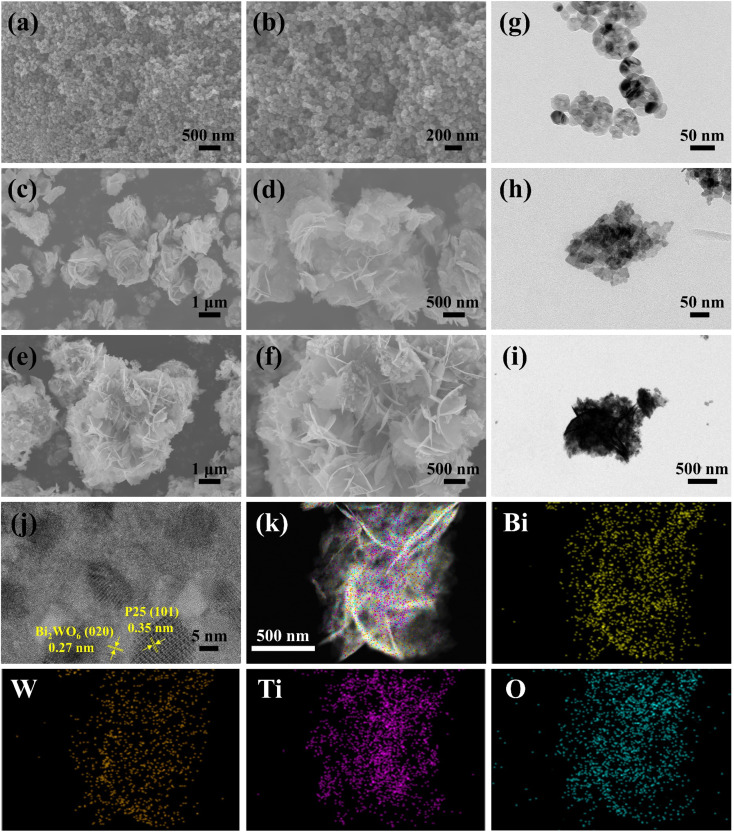
(a–f) SEM images, and (g–i) TEM images of P25, BWO and P25/BWO-20 heterojunction; (j) HRTEM image, and (k) STEM and the corresponding elemental mapping images of P25/BWO-20 heterojunction.

SEM and TEM analyzed the morphology and microstructure of the samples. As shown in Fig. 2a–d, pure P25 was a nanoparticle with agglomerated morphology, and BWO was a nanosheet structure with a hierarchical structure. The formation of this hierarchical structure may be due to the anisotropy and self-assembly of BWO nanosheets. For the P25/BWO-20 heterojunction, many P25 nanoparticles can be uniformly distributed in the BWO nanosheets (Fig. 2e and f). From the high-resolution electron microscopy images (Fig. 2j), it can be seen that the two components, P25 and BWO, were in close contact, forming a clear heterojunction interface rather than a simple physical mixing. The lattice spacing of 0.27 nm was assigned to the (020) crystal plane of BWO,^43^ and the lattice spacing of 0.352 nm corresponded to the (101) plane of anatase TiO_2_.^44^ Elemental EDS spectral analysis (Fig. 2k and S1†) revealed the elemental composition and distribution in the P25/BWO-20 heterojunction, and the elemental profiles of Bi, W, Ti, and O were uniformly distributed, indicating that the P25 nanoparticles are uniformly distributed in the nanocomposites. These results indicated that we successfully constructed P25/BWO heterojunction.

### 3.3. Surface and composition analysis

**Fig. 3:**
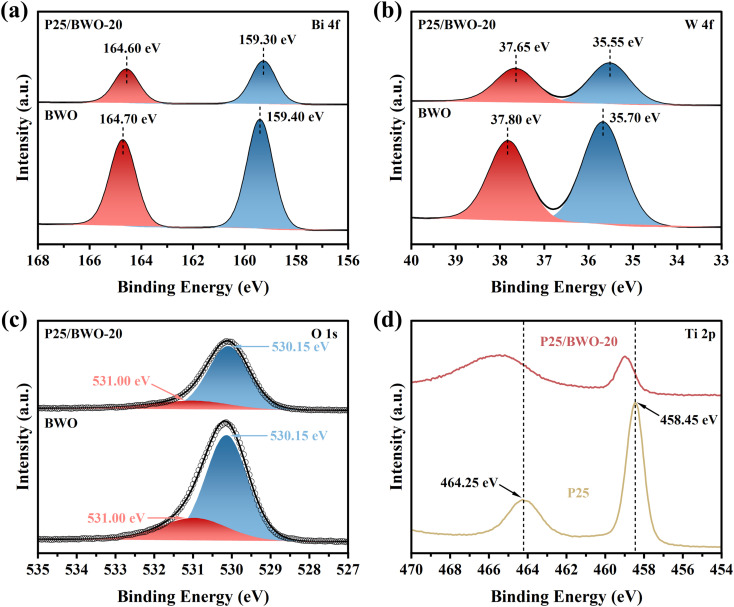
High-resolution XPS spectra of (a) Bi 4f, (b) W 4f, and (c) O 1s in P25/BWO-20 heterojunction and BWO, and (d) Ti 2p in P25/BWO-20 heterojunction and P25.

The chemical composition and surface valence states of P25, BWO, and P25/BWO-20 heterojunction were investigated by XPS. Full-spectrum analysis of different samples (Fig. S2†) showed that the P25/BWO-20 heterojunction was mainly composed of four elements, Bi, W, Ti, and O. In order to elucidate the interfacial interaction between P25 and BWO, the high-resolution XPS spectra of different samples of Bi 4f, W 4f, Ti 2p, and O 1s were also investigated. The Bi 4f spectrum of BWO (Fig. 3a) had two peaks at 159.40 and 164.70 eV, which were attributed to Bi 4f_7/2_ and Bi 4f_5/2_ of the Bi^3+^ ion, respectively. As displayed in Fig. 3b clearly, the peaks at 35.70 and 37.80 eV of the W 4f spectrum corresponded to W 4f_7/2_ and W 4f_5/2_ of the W^6+^ ion in BWO, respectively.^45^ As shown in Fig. 3d, the characteristic peaks with binding energies of 458.45 and 464.25 eV were designated as Ti 2p_3/2_ and Ti 2p_1/2_, respectively, indicating the presence of Ti^4+^.^21^ Compared to the pristine BWO, the P25/BWO-20 heterojunction's binding energies were negatively shifted by 0.1 and 0.15 eV for Bi 4f and W 4f, respectively. Specifically, the negative shift in binding energy indicates an increase in electron density.^46^ Therefore, the migration path of electrons in the composite can be determined by shifting the XPS binding energy. In this case, the negative shifts of the binding energy of Bi 4f and W 4f indicated that BWO acts as an electron acceptor in the P25/BWO-20 heterojunction. In order to balance the electron redistribution in the P25/BWO-20 heterojunction, Ti in P25 will be electron deficient. The latter was confirmed by the positive shift of the Ti 2p binding energy in the P25/BWO-20 heterojunction, which implied a decrease in electron density. In addition, the O 1s spectrum of the P25/BWO-20 heterojunction (Fig. 3c) can be divided into two peaks at 530.15 and 531.00 eV, which were attributed to the lattice oxygen and surface hydroxyl group, respectively.^47,48^ By observing the binding energy, the results indicated that the photogenerated electrons migrate from P25 to BWO during photoexcitation, where P25 was the electron donor and BWO was the electron acceptor. The detailed mechanism of the electron transfer pathway in the P25/BWO-20 heterojunction will be further elaborated later.

### 3.4. Optical properties and energy band structure

**Fig. 4:**
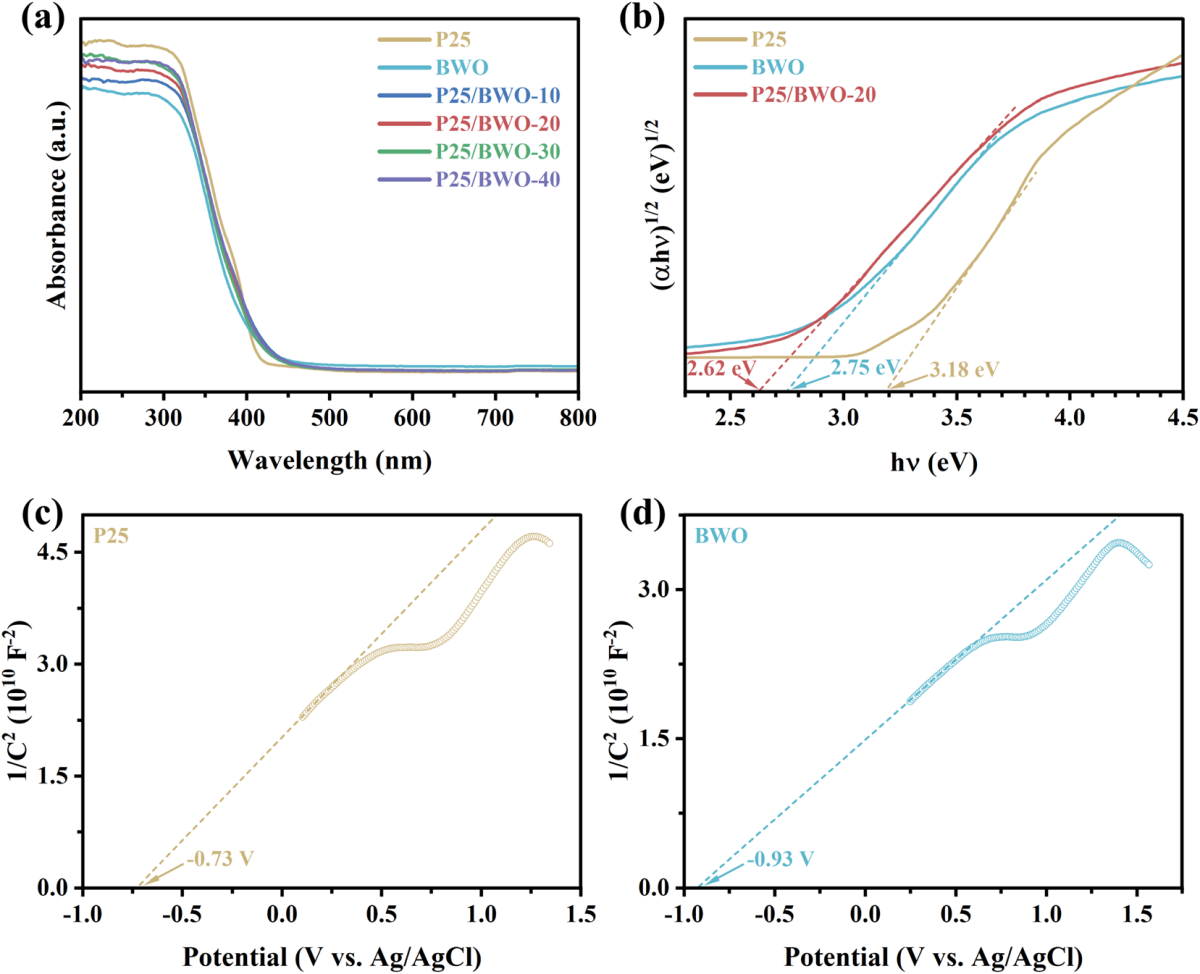
(a) UV-vis DRS, and (b) Tauc plots of P25, BWO and P25/BWO-20 heterojunction; (c and d) Mott–Schottky plots of P25 and BWO.

The optical absorbance and energy band structure of P25, BWO, and P25/BWO nanocomposites with different contents were investigated by UV-vis DRS. As shown in Fig. 4a and S3,† P25/BWO-20 heterojunction exhibits a feature intermediate between its constituent components, and the absorption edges of P25 and BWO are 414 nm and 449 nm, respectively. Compared with BWO, the absorption edges of the P25/BWO-20 heterojunction showed a slight red-shift and steep curve due to the band gap jump between P25 and BWO. The visible absorption range was extended to 464 nm, indicating that the synthesized nanocomposites had significantly higher light utilization and could absorb solar energy more efficiently and generate electron–hole pairs for photocatalytic CO_2_ reduction. Furthermore, based on the Kubelka–Munk equation:^49^

1 *α*ℏ*ν* = *k*(ℏ*ν* − *E*_g_)^*n*/2^

where *α* is the absorption coefficient, ℏ is Planck's constant, *ν* is the incident light frequency, *k* is a constant, *E*_g_ is the bandgap energy, and the value of *n* for the indirect bandgap semiconductors is 1. Fig. 4b shows the relationship between (*α*ℏ*ν*)^1/2^ and ℏ*ν*. It was inferred that the band gap widths of P25, BWO, and P25/BWO-20 heterojunction were 3.18, 2.75, and 2.62 eV, respectively. In summary, the enhanced light absorption and narrower forbidden bandwidths were favorable to improve the photocatalytic activity.

Mott–Schottky analysis was performed by impedance-potential testing in a typical three-electrode system to determine the semiconductor type and electronic energy band structure of the sample, where Mott–Schottky diagrams were plotted according to the following equations:^50^

For p-type semiconductors:2
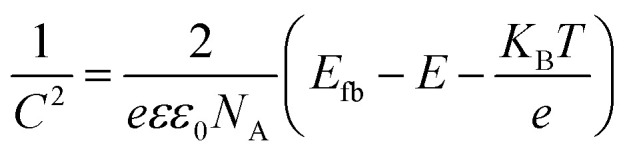


For n-type semiconductors:3
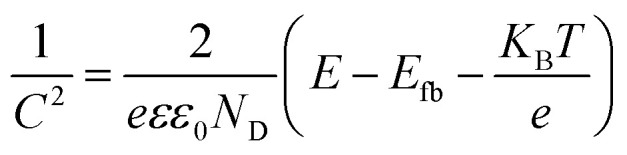
where *C* is the capacitance of the space charge layer, *e* is the electronic charge (*e* = 1.602 × 10^−19^ C), *ε* is the dielectric constant of the semiconductor, *ε*_0_ is the permittivity in vacuum (*ε*_0_ = 8.8510^−14^ F cm^−1^), *N*_A_ and *N*_D_ are the numbers of acceptors and donors in p-type and n-type semiconductors, respectively, *E* is the applied potential, *E*_fb_ is the flat-band potential, *K*_B_ is the Boltzmann's constant, and *T* is the absolute temperature. Therefore, *E*_fb_ can be estimated by extrapolating the Mott–Schottky curves to the *x*-axis (*i.e.*, 1/*C*^2^ = 0).

As shown in Fig. 4c and d, the slopes of both P25 and BWO are positive, indicating that they were both n-type semiconductors. The experimental potential measured relative to the Ag/AgCl reference electrode can be converted to the standard hydrogen electrode potential by the Nernst equation:^51^

4 *E*_fb_(*vs.* NHE) = *E*_fb_(*vs.* Ag/AgCl) + *E*^0^_Ag/AgCl_ + 0.059pH

where *E*_fb_ (*vs.* NHE) is the standard hydrogen electrode potential, *E*_fb_ (*vs.* Ag/AgCl) is the experimental potential measured against the Ag/AgCl reference electrode and *E*^0^_Ag/AgCl_ is the standard potential of Ag/AgCl at 298 K (0.1976 V). Considering that the conduction band edge of n-type semiconductors is generally 0.3 V lower than the standard hydrogen electrode potential,^52^ the conduction bands of P25 and BWO were calculated to be −0.42 and −0.62 eV, respectively, and the valence bands can be determined by eqn (5):^53^

5 *E*_VB_ = *E*_CB_ + *E*_g_

The results showed that the valence bands of P25 and BWO were 2.76 and 2.13 eV, respectively.

### 3.5. Carriers' separation

**Fig. 5:**
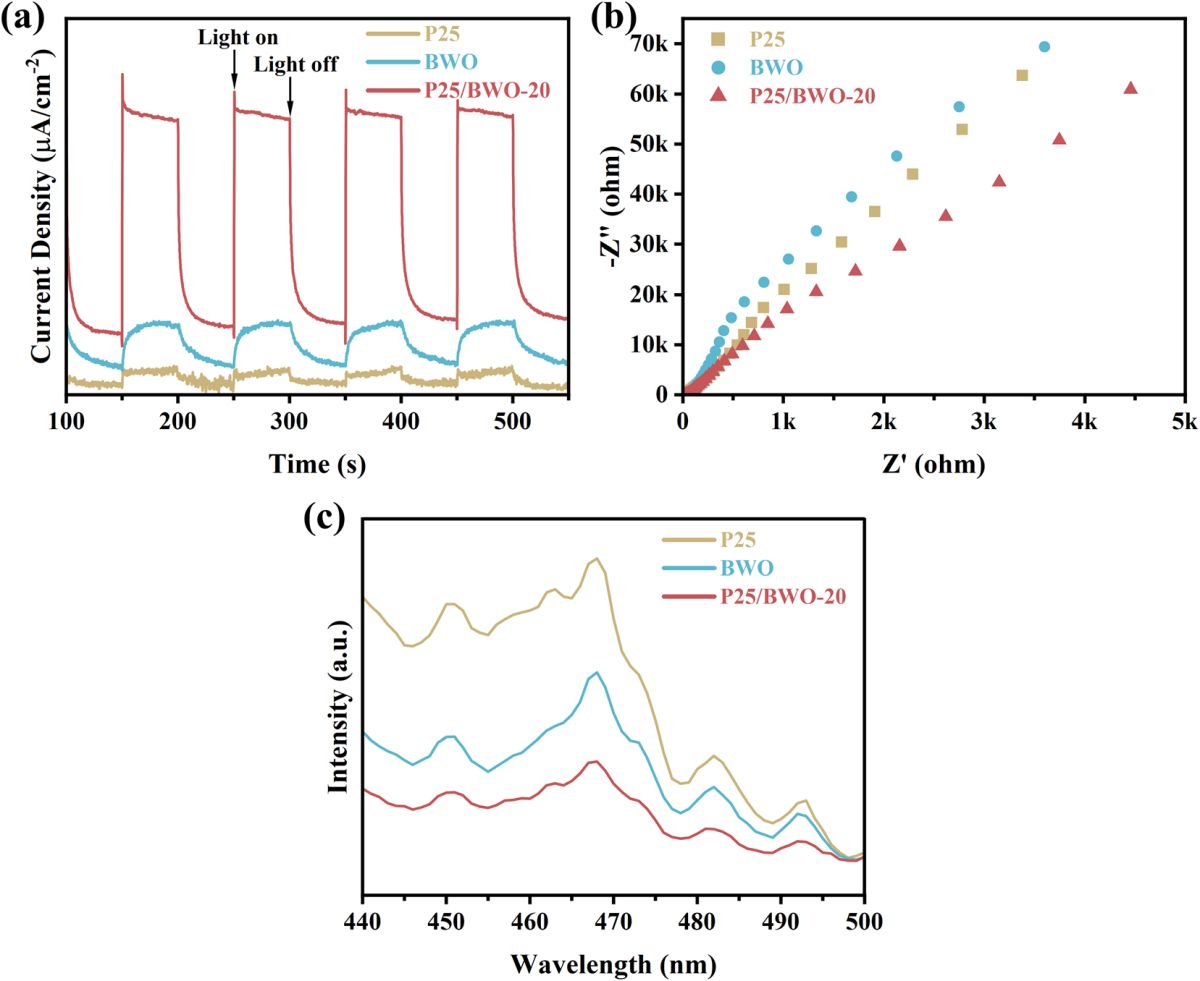
(a) Transient photocurrent, (b) EIS plots, and (c) PL spectra of P25, BWO and P25/BWO-20 heterojunction.

In addition to the semiconductor energy band structure, the effective electron–hole pair separation is also one of the critical factors affecting photocatalytic efficiency. The samples' charge separation and transfer capability were investigated using transient photocurrent response spectra and AC impedance spectra. Firstly, the transient photocurrent response of the samples was examined by the on–off cycle of simulated solar irradiation. Briefly, photogenerated electrons were transferred to the back contact to generate the photocurrent response.^54,55^ Simultaneously, holes migrate to the interface between the photocatalyst and the electrolyte and are then captured by the reduced species in the electrolyte under illumination. Since the electrolyte and electrodes used are the same each time, the instantaneous photocurrent intensity exhibited by the sample reflects the efficiency of photogenerated carrier generation and separation.^56,57^ As shown in Fig. 5a, the P25/BWO-20 heterojunction exhibited a higher photocurrent response than pure P25 and BWO, indicating that the P25/BWO-20 heterojunction had better charge separation and transfer efficiency. It was worth mentioning that the photocurrent intensities of the samples were consistent with their results in photocatalytic CO_2_ reduction experiments, indicating that separating electron–hole pairs is a decisive factor in photocatalytic performance. In addition, in the absence of light, the photocurrent intensity of the samples was decaying, which can be attributed to the slow discharge of the accumulated charge in the middle layer of the electrodes.^58^ The AC impedance spectra can reflect the charge transfer kinetics at the electrode/electrolyte interface. The smaller the arc radius of the curve, the lower the reaction electron transfer resistance and the faster the photogenerated carrier transfer rate, leading to a more superior interfacial charge transfer process.^59,60^ As shown in Fig. 5b, the P25/BWO-20 heterojunction had the smallest arc radius, indicating a faster charge transfer rate at the interface. The results of transient photocurrent response and AC impedance spectra indicated that the presence of heterojunction in the P25/BWO nanocomposites facilitates the electron transfer process, which effectively inhibited the recombination of electron–hole pairs and significantly improved the photocatalytic activity of the samples. Steady-state photoluminescence spectra further verified the charge separation and transferred efficiency of the samples after excitation at 308 nm. As shown in Fig. 5c, pure P25 and BWO had strong fluorescence emissions at ∼468 nm, indicating a severe recombination of carriers. However, the fluorescence intensity significantly decreased after forming P25/BWO heterojunction, indicating that the formation of a heterojunction can effectively suppress the recombination of electron–hole pairs and facilitate the photocatalytic CO_2_ reduction.^61^ Therefore, these results suggested that the construction of heterojunction is an effective strategy to improve photocatalytic activity.

### 3.6. Photocatalytic activities

**Fig. 6:**
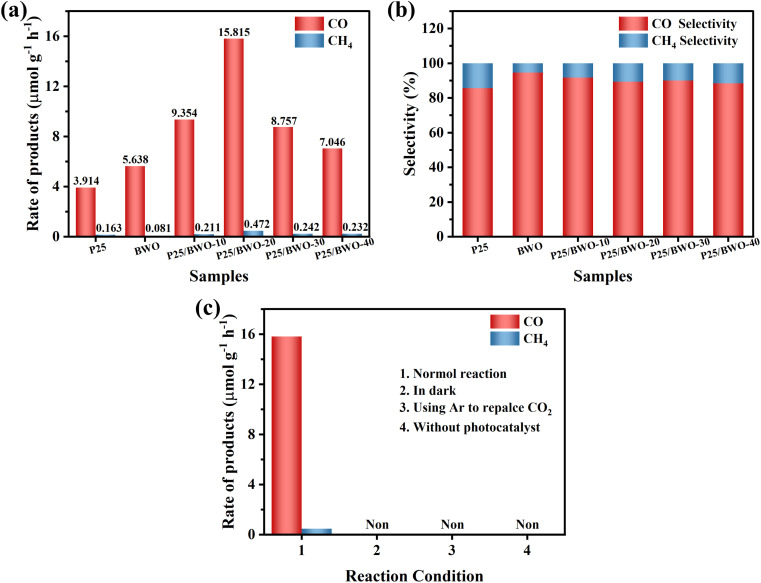
(a) Photocatalytic activity test, and (b) selectivity of the as-prepared samples; (c) photocatalysis under different conditions performance chart of P25/BWO-20 heterojunction.

The photocatalytic CO_2_ reduction reaction (CO_2_RR) was carried out in a closed quartz glass reaction apparatus without adding any co-catalysts, photosensitizers, or sacrificial agents, and the photocatalytic reactions of all samples were studied in the gas–solid system under simulated solar irradiation. As shown in Fig. 6a, pure P25, and BWO photocatalytic activities were not high due to poor charge separation and severe charge recombination. Meanwhile, the main products of both P25 and BWO were CO. When the P25 nanoparticles and BWO nanosheets were compounded, the P25/BWO nanocomposites exhibited stronger photocatalytic activity. The maximum yield of CO reached 15.815 μmol g^−1^ h^−1^ on the P25/BWO nanocomposites, which was 4.04 and 2.80 times higher than that of P25 and BWO, respectively, indicating the formation of a heterojunction between the P25 and BWO interfaces. In addition, the content of P25 in the P25/BWO nanocomposites was optimized, and the results showed that the photocatalytic activity of the samples was enhanced with the increase of P25 content in the nanocomposites, showing a volcanic trend, and the optimal content of P25 in the nanocomposites could be concluded to be 20%. Optimizing the content of P25 can improve the light absorption efficiency of the P25/BWO heterojunction. However, the excessive P25 will block the catalytic active center of the heterojunction and reduce the photocatalytic activity. The product selectivity can observe the effect of the loading of P25 in the nanocomposites on the reduction of CO_2_. Based on the number of electrons transferred to produce each reduction product (2e^−^ for CO and 8e^−^ for CH_4_), the selectivity of CO is defined as follows:^62^6
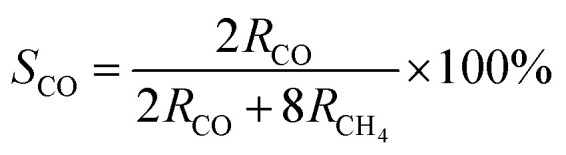
where *S*_CO_ is the selectivity of CO, *R*_CO_ and *R*_CH_4__ are the yields of CO and CH_4_, respectively. Fig. 6b shows the calculated selectivity of CO and CH_4_ for all samples. The selectivity of pure P25 and BWO for CO was 85.69% and 94.58%, respectively. For the P25/BWO nanocomposites, the selectivity of CO was ∼90%. The results indicated that P25/BWO nanocomposites have a high selectivity for CO, mainly due to the easy desorption of CO and the need for more complex intermediates for CH_4_ generation. In addition, a series of control experiments (Fig. 6c) were performed in the absence of light, CO_2_, or photocatalyst, where the product yields were negligible, which could indicate that light is the driving force of the CO_2_ reduction process and that the carbon source of the product CO was derived from CO_2_.

### 3.7. Photocatalytic mechanism of CO_2_ reduction

**Scheme 2:**
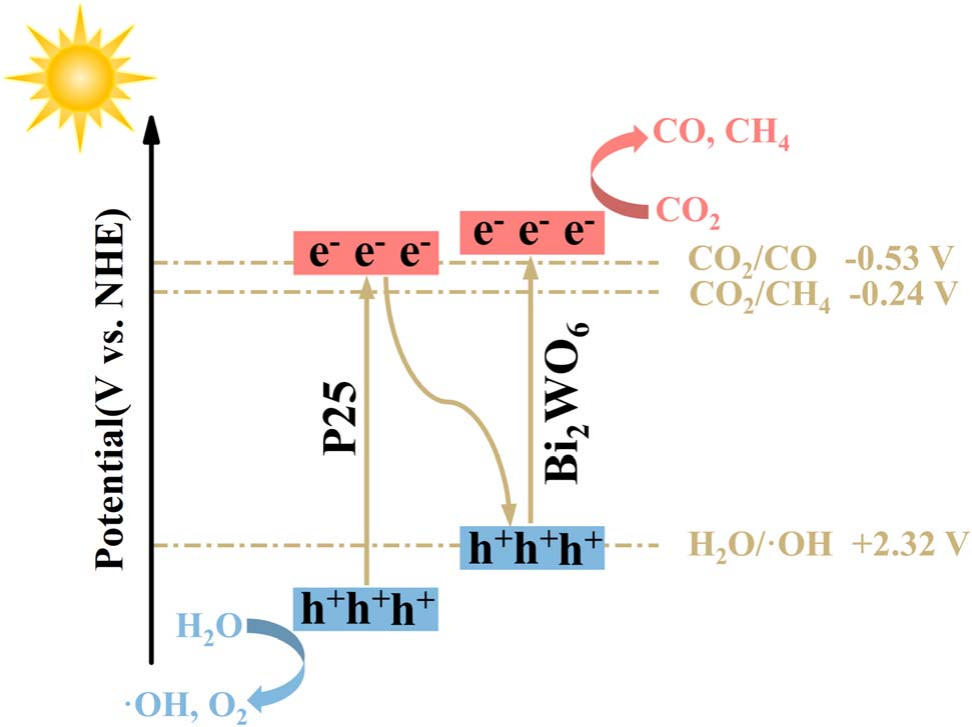
Proposed mechanism of CO_2_ photoreduction due to heterojunction formation within over P25/BWO nanocomposites.

Based on the results above, the charge transfer path at the P25/BWO heterojunction interface may follow the Z-scheme mechanism. As shown in Scheme 2, under simulated solar irradiation, P25 and BWO produce photogenerated electrons and holes in their conduction band (CB) and valence band (VB) positions, respectively, under photoexcitation, where the photogenerated electrons in the CB of P25 will be transferred to the VB of BWO and recombine with the holes produced in the VB. In contrast, the holes in the VB of P25 will react with water (H_2_O) to form hydroxyl groups (·OH) and protons (H^+^). The photogenerated electrons are then retained in the CB of BWO, which can convert CO_2_ into CO and CH_4_. This Z-scheme charge transfer mechanism enables the P25/BWO nanocomposites to have an efficient charge separation capability, which significantly improves the photocatalytic CO_2_ reduction performance of the P25/BWO nanocomposites. It is well known that the different structural characteristics and reaction conditions of catalysts lead to different reaction products. In the photocatalytic CO_2_ reduction reaction (CO_2_RR), eight electrons (8e^−^) are required to generate one CH_4_ molecule, while only two electrons (2e^−^) are required to generate one CO molecule. Therefore, it will be more challenging to generate CH_4_. Converting CO_2_ to CO may be more kinetically advantageous in our photocatalytic reaction system. Therefore, in the present work, the P25/BWO nanocomposites can convert CO_2_ into CO with very high selectivity.

**Fig. 7:**
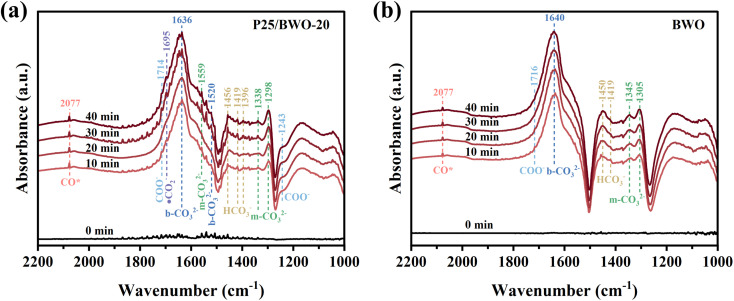
(a and b) *In situ* DRIFTS spectra of surface adsorbed CO_2_ species and photocatalytic CO_2_ reduction intermediates of P25/BWO-20 heterojunction and BWO.

In order to explore the process and intermediates of efficient and selective reduction of CO_2_ to CO for photocatalysts in more detail, *in situ* diffuse reflectance infrared Fourier transform spectra (DRIFTS) was carried out. Fig. 7a, b and S4† showed the carbon-based groups' dynamic changes on the photocatalysts' surface within 0–40 min of illumination. Several characteristic peaks attributed to bicarbonate (HCO_3_^−^, 1456, 1419, and 1396 cm^−1^), monodentate carbonate (m-CO_3_^2−^, 1559, 1338, and 1298 cm^−1^), bidentate carbonate (b-CO_3_^2−^, 1636 and 1520 cm^−1^), carboxylate (COO^−^, 1714 and 1243 cm^−1^), and active ·CO_2_^−^ intermediates (1695 cm^−1^) were detected. Hence, the photocatalytic CO_2_ reduction can be significantly enhanced due to those important intermediates. In comparison, the signal changes of carbonate and bicarbonate species during reaction processes in the spectra for P25 and BWO are much weaker, indicating a poor ability of CO_2_ conversion under the illumination of P25 and BWO. Moreover, the significant peak absorbance of CO* (2077 cm^−1^) supports the formation of CO during photocatalytic CO_2_ reduction.^7,63–65^

## 4 Conclusions

In summary, we prepared P25/BWO nanocomposites by a one-step *in situ* hydrothermal method for efficient photocatalytic CO_2_ reduction. Their respective interlaced energy band structures promote charge separation and inhibit charge recombination. The P25/BWO nanocomposites exhibited strong synergy between the semiconductor components, leading to good activity and selectivity for the photoreduction of CO_2_ to CO under simulated solar irradiation. The samples had the highest activity when the content of P25 in the nanocomposites is 20%, when maximization of the heterojunction interface may be achieved. The CO yield of the P25/BWO-20 heterojunction reached 15.815 μmol g^−1^ h^−1^, which was 4.04 and 2.80 times higher than that of pure P25 and BWO, respectively. In addition, the P25/BWO-20 heterojunction showed a high selectivity of 89.33% for CO, mainly due to the easy desorption of CO and the need for more complex intermediates for CH_4_ generation. This work presents an effective strategy to facilitate charge separation by constructing Z-scheme heterojunction. This strategy opens up new opportunities for designing and developing other novel Bi_2_WO_6_-based photocatalysts.

## Ethical approval

The experimental protocols were not involved by any animals or human care.

## Data availability

The datasets used and/or analyzed during the current study are available from the corresponding author on reasonable request.

## Author contributions

D. Liu: Investigation, Data Curation, writing – original draft; M. Zeng: validation, formal analysis, writing – review & editing; Z. Li: methodology; Z. Zhu: resources; Y. Chen: data curation; K. Thummavichai: supervision; O. Ola: investigation, formal analysis; N. Wang: project administration; Y. Zhu: conceptualization, funding acquisition.

## Conflicts of interest

The authors have no conflicts of interest to declare.

## Supplementary Material

RA-013-D3RA00418J-s001
