# Supplementary material for: Interfacial construction of P25/Bi2WO6 composites for selective CO2 photoreduction to CO in gas–solid reactions
Source: RSC Adv. 2023 Mar 14;13(13):8564–76. doi: 10.1039/d3ra00418j (PMC10013126; doi:10.1039/d3ra00418j)
Supplement: RA-013-D3RA00418J-s001 [file RA-013-D3RA00418J-s001.pdf]

Electronic Supplementary Material (ESI) for RSC Advances.
This journal is © The Royal Society of Chemistry 2023

Supplementary material for

Interfacial construction of P25/Bi_2WO_6 composites for selective CO_2 photoreduction to CO in Gas-Solid reaction

3. Results and discussion

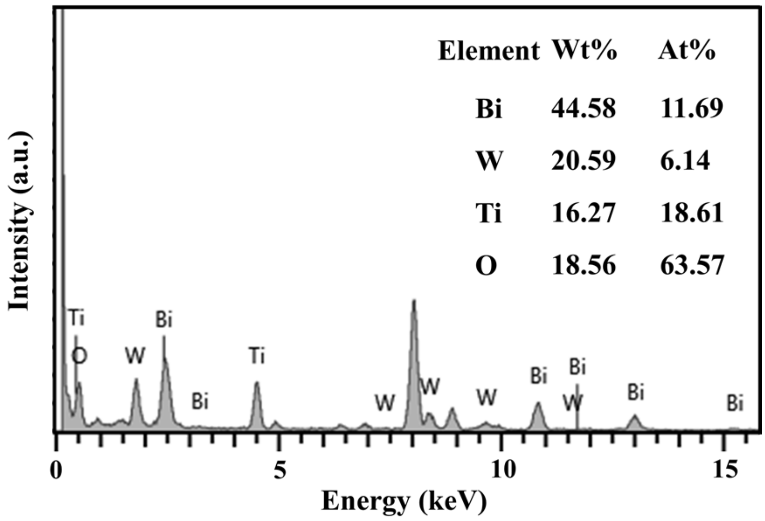

Fig. S1. EDS spectra of P25/BWO-20 heterojunction.

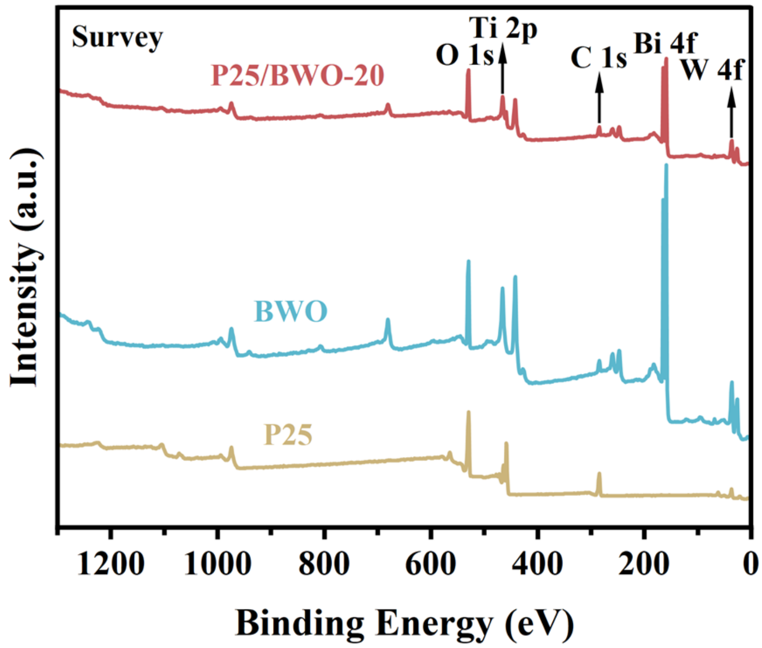

Fig. S2. XPS survey spectra of P25, BWO and P25/BWO-20 heterojunction.

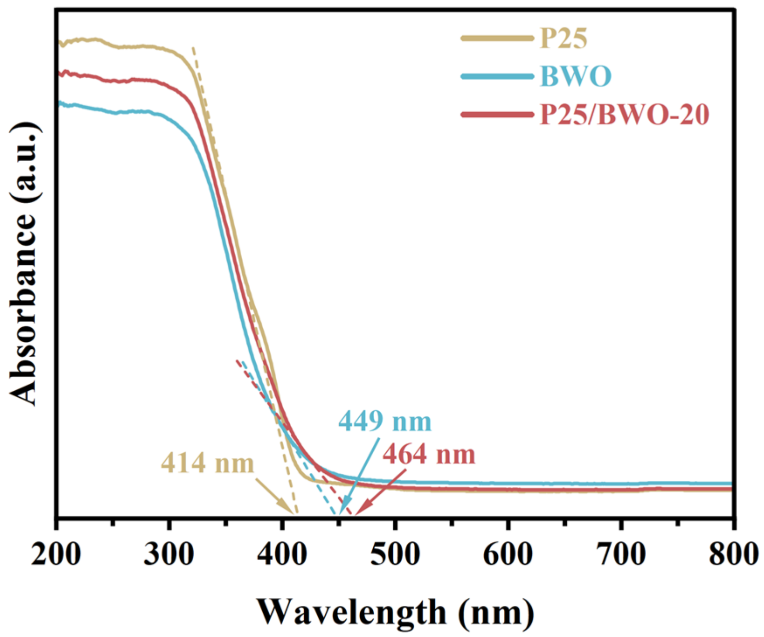

Fig. S3. UV-vis DRS of the as-prepared samples.

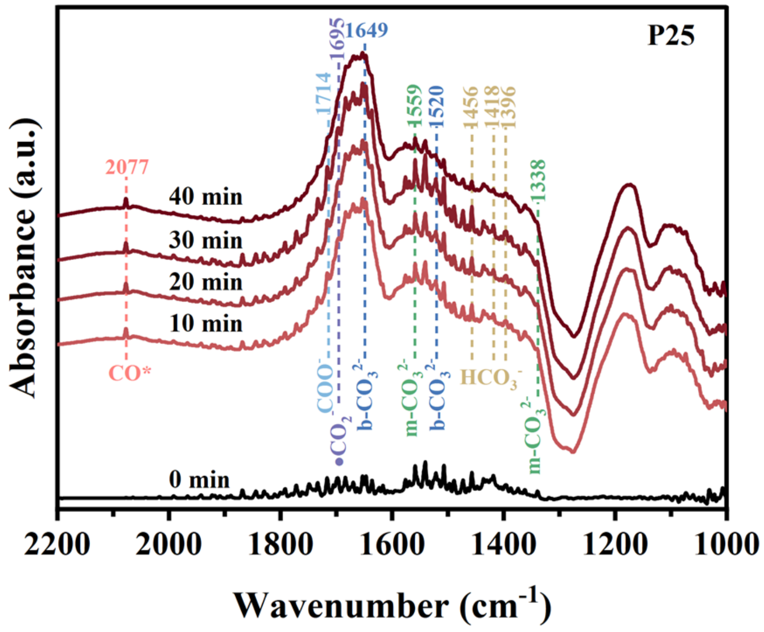

Fig. S4. In-situ DRIFTS spectra of surface adsorbed CO_2 species and photocatalytic CO_2 reduction intermediates of P25.
